# Effect of religious fatalism and concern about new variants on the acceptance of COVID-19 vaccines

**DOI:** 10.3389/fpsyt.2023.1071543

**Published:** 2023-03-02

**Authors:** Oscar Mamani-Benito, Rosa Farfán-Solís, Mariné Huayta-Meza, Madona Tito-Betancur, Wilter C. Morales-García, Edison Effer Apaza Tarqui

**Affiliations:** ^1^Facultad de Derecho y Humanidades, Universidad Señor de Sipán, Chiclayo, Peru; ^2^Facultad de Enfermería, Universidad Nacional del Altiplano, Puno, Peru; ^3^Facultad de Ciencias Empresariales, Universidad Peruana Unión, Juliaca, Peru; ^4^Facultad de Derecho, Universidad Tecnológica del Peru, Arequipa, Peru; ^5^Unidad de Salud, Escuela de Posgrado, Universidad Peruana Unión, Lima, Peru; ^6^Maestría en Ciencia de los Datos, Escuela de Posgrado, Universidad Ricardo Palma, Lima, Peru

**Keywords:** fatalism, religiosity, COVID-19 vaccine, vaccine acceptance, Peru

## Abstract

**Introduction:**

To protect public health, it is important that the population be vaccinated against COVID-19; however, certain factors can affect vaccine acceptance.

**Objective:**

The objective of this study was to determine whether religious fatalism and concern about new variants have a significant effect on the acceptance of COVID-19 vaccines.

**Methodology:**

An explanatory study was conducted with 403 adults of legal age captured through non-probabilistic convenience sampling in vaccination centers in the 13 health networks of the Regional Health Directorate of Puno, Peru. Data were collected through a brief scale of religious fatalism, a scale of acceptance of vaccines against COVID-19 and a scale of concern about a new variant of COVID-19.

**Results:**

The proposed model obtained an adequate fit. There was a negative effect of religious fatalism on vaccine acceptance, a positive effect of fatalism on vaccine rejection, a positive effect of concern about new variants on the acceptance of vaccines, and a positive effect of concern about new variants on vaccine rejection.

**Conclusion:**

These findings provide evidence for the usefulness of considering both religious fatalism and concern about new variants affect the intention to receive the COVID-19 vaccine in adults in southern Peru.

## 1. Introduction

Faced with the rapid spread of SARS-CoV-2 (1), scientists were forced to speed up the vaccine development process (2, 3). Thanks to unprecedented scientific and financial support, they were able to successfully produce prominent results, making it possible for clinical trials to be conducted in record time (4). It was expected that the population would have full confidence and security in receiving the recommended doses (3), however, a large portion of it questioned the value of COVID-19 vaccines, influenced by conspiracy theories (5, 6) and conflicting religious beliefs (7). Additionally, the emergence of new variants further increased uncertainty among those who intended to be vaccinated, leading some to believe that only certain vaccines from a select few laboratories were effective enough in protecting against the effects of these new variations. It is important to note that the references cited are not clear and it is not possible to verify their accuracy or relevance to the statement (8).

Regarding the acceptance of COVID-19 vaccines, available scientific literature shows that in countries such as Vietnam, India, China, Denmark, South Korea, Serbia, Croatia, France, Lebanon, and Paraguay, the population demonstrated acceptance of vaccines (9); however, in many other countries, especially in Latin America, there is still an unfavorable attitude, partly due to the role played by the media (10), irrational beliefs and fatalistic ideas stemming from culture, and the lack of scientific information about the importance of vaccines (7, 11). This scenario has sparked interest among various researchers to discover the predictors of the intention to get vaccinated (12), as is the case of the present study, in which the authors focus on the concern for infection amid a new variant of COVID-19 and religious fatalism.

As for the concern about new variants of SARS-CoV-2, this phenomenon has caused great alarm in the scientific community and the general population (13), as at one point it was not clear the level of lethality and possible health sequels (14). On this topic, it is known that viruses are constantly changing through mutation (15), so some variants tend to emerge and then disappear, while others persist; and with regard to the mutations of SARS-CoV-2, to date of this report various variants of the virus have been documented (16), in response, scientists are conducting virological and epidemiological research to evaluate in depth the transmissibility, severity, risk of reinfection and the response of antibodies to these variations (17); meanwhile, the population remains alert and showing concern about the possibility of reinfection or experiencing unknown sequels (18).

Regarding religious fatalism, the literature reveals how controversial conflicting religious beliefs are in relation to the origin of SARS-CoV-2 (19). These beliefs clearly challenge public health, leading to the assumption that everything happens by the will of God, that life and death are matters of destiny, even that COVID-19 represents a deviation from faith, an idea that is contrary to what is actually healthy, as religion plays an active role in health; in this case, religious beliefs should encourage the use of health services (20). Accordingly, recent studies have framed the construct as religious fatalism in the face of COVID-19 (21, 22). Thus, fatalistic beliefs lead to thinking that one is not in control of their actions (23), leading to the assumption that fate cannot be changed and that the events of life are beyond one’s control (24). This view is why this construct is interpreted as the belief that health outcomes are inevitable and/or determined by a higher power, that is, God (25). In the context of COVID-19, this construct translates to the perception that the presence of the new coronavirus is a predetermined fact and that both infection or possible death from the virus occurs by divine will and punishment. This conception is enhanced by anti-vaccine religious leadership (26).

Based on the reviewed scientific literature, at the beginning of the health emergency, fatalism has caused a part of the population to perceive the coronavirus as a death sentence, which has generated reluctance to perform recommended preventive behaviors, such as handwashing and social distancing (27). With the emergence of vaccines, these fatalistic ideas combined with conflicting religious beliefs have caused a large part of the population to doubt the efficacy of vaccines against COVID-19 (28), because in a large part of the population, the belief that the body is a temple that should not be profaned was greater, compared to the thought that scientific advances are essential to preserve the health of the community (29). In addition, the emergence of new variants complicated the COVID-19 vaccination plan, as it is known that COVID-19 vaccines had to be updated, but emerging variants and volatile immune reactions conditioned how the new injections should be. For example, existing vaccines based on the version of the SARS-CoV-2 virus that emerged in Wuhan, China, do not match the current strains of Omicron. As a result, vaccines now only offer short-term protection against infection, but appear to be resisting severe diseases (30).

As observed, there is evidence to assume functional relationships between religious fatalism and vaccine acceptance, or, between concern for new variants and the intention to vaccinate, however, there are still very few studies that analyze under explanatory models the determinants of the intention to vaccinate; in response, the investigators of the present study propose the following hypotheses:

### 1.1. Hypotheses

Under the premise that it is necessary to investigate the factors that inhibit preventive behaviors (27), the researchers propose the following research hypotheses:

H1: The greater the religious fatalism is, the lower the acceptance of vaccines against COVID-19 is.

H2: The greater the concern about a new variant is, the lower the rejection of vaccines against COVID-19 is.

H3: The greater the religious fatalism is, the greater the rejection of vaccines against COVID-19 is.

H4: The greater the concern about a new variant is, the greater the acceptance of vaccines against COVID-19 is.

Based on what has been proposed, the following research objectives are proposed:

• First: to determine whether religious fatalism generates less acceptance of COVID-19 vaccines.
• Second: to determine if a greater concern for a new variant generates rejection of COVID-19 vaccines.
• Third: Determine if a higher religious fatalism generates rejection of COVID-19 vaccines.
• Fourth: Determine if a greater concern for a new variant generates acceptance of COVID-19 vaccines.

## 2. Materials and methods

### 2.1. Design

Cross-sectional explanatory study (31).

### 2.2. Participants

Under non-probabilistic convenience sampling, 403 adults of both genders (61% women) between 18 and 91 years of age (ME = 35.22, SD = 12.01) participated. Participants were recruited from the vaccination centers of the 13 health networks of the Regional Directorate of Health of Puno, Peru. A total of 42.7% were married, 26.8% were cohabiting, 24.8% were single, and to a lesser extent, widowed and divorced (5.7%). Most of them had a higher level of education (73.4%), 48.1% had a dependent job, 47.6% were independently employed and 4.2% had both types of jobs. A total of 45.2% attended the vaccination center to receive their dose for reasons of travel (45.2%), work (30%), and health (24.8%). At the time of the survey, 46.7% had received their third dose (booster), 36.2%, their second dose, 11.2%, none, 5%, their first dose, and 1%, their fourth dose (booster).

### 2.3. Instruments

#### 2.3.1. Concern about a new variant of COVID-19

This issue was evaluated through a scale created for Peruvian adults (32) adapted to the context of the new variants by Esteban et al. (33). It is composed of five items distributed in a single factor; in addition, the responses are scored on a 4-point Likert scale: never or rarely, sometimes, often, and almost all the time. The scale showed adequate psychometric properties in 407 adults from the three regions of Peru; content validity (*V* > 0.70) and construct validity were confirmed with confirmatory factor analysis (with gender invariance), convergent and divergent validity were confirmed with Pearson correlation analysis, and reliability were confirmed with the Omega coefficient (ω > 0.80).

#### 2.3.2. Perception of the acceptance of vaccines against SARS-CoV-2

This issue was evaluated with a scale created by Mejia et al. (34) for Peruvian adults. It consists of 11 items distributed in two factors (acceptance and rejection), with responses scored on a 5-point Likert scale: completely disagree, disagree, neither disagree nor agree, agree, and completely agree. This version showed adequate psychometric properties in 3,000 citizens of 24 departments of Peru, where Aiken’s V was greater than 0.70, the construct validity was corroborated with confirmatory factor analysis, and the reliability was confirmed with Cronbach’s alpha (>0.80).

#### 2.3.3. Religious fatalism

It was evaluated with a scale translated and adapted by Mamani-Benito et al. (7), who based his study on a scale designed by Franklin et al. (25). It consists of nine items distributed in two factors (Divine provision and plan destined) with responses scored on a 4-point Likert scale: Never or rarely, Sometimes, Often, Almost all the time. The scale showed adequate psychometric properties in 764 adults from the three regions of Peru; where Aiken’s V was greater than 0.70, the construct validity was corroborated with confirmatory factor analysis, and the reliability was confirmed with Cronbach’s alpha (0.89; CI 0.95%: 0.79–0.82).

### 2.4. Procedure

The study was conducted between January and February of 2022, in the middle of the third wave of infections. A questionnaire was developed to capture demographic information and included the corresponding scales. Data were collected with the questionnaire and by monitoring by one of the researchers. To recruit participants, the researchers contacted the vaccination centers authorized in the 13 DIRESA health networks of Puno, Peru. There, they had the support of health personnel who were previously trained in administering the surveys; specifically, the instruments were applied after inoculation with the respective vaccine (observation period). Informed consent was requested from all participants, who were informed of the confidential handling of the data.

### 2.5. Data analysis

Structural equation modeling (SEM) was applied. First, in the model specification phase, it was established to include religious fatalism and concern for a new variant as variables that explain vaccine acceptance. In the second phase, before collecting the data, it was determined whether the model is correctly identified, for this, the degrees of freedom (gl) were calculated, assuming that gl ≥ 0 demonstrates an identified or over-identified model. Thirdly, in the model estimation phase, the MLR estimator was chosen, ideal for numerical variables, having the property of being robust against deviations of inferential normality (35). Fourthly, the evaluation of the model was performed through goodness-of-fit indices, such as the comparative fit index (CFI), the mean square approximation error (RMSEA) and the standardized root mean square residual (SRMR), for which the values of CFI > 0.90 (36), RMSEA < 0.080, and SRMR < 0.080 (37) were used. Fifthly, the possibility of re-specifying the model was analyzed, which, in this research, was not necessary.

As for the reliability analysis, it was carried out through the internal consistency method (Cronbach’s Alpha). All calculations were performed using the “R” software in version 4.1.2, using the “lavaan” and “semploth” libraries in version 0.6-10 (38).

### 2.6. Ethical considerations

The research was approved by the research ethics committee of the Universidad Peruana Unión (N° 2022-CEUPeU-025).

## 3. Results

The scores of the study variables were scaled with values between 0 and 30 to facilitate visualization. This procedure did not affect the values of the correlations between the variables. Table 1 shows the descriptive results, such as asymmetry (A) and kurtosis (K). In addition, the absolute values of the correlation results for the study variables are between 0.12 and 0.90. This table also shows the alpha coefficients of internal consistency, which were found to be between 0.70 and 0.92.

**TABLE 1:** Descriptive data and correlations of the study variables.

Variables	*M*	SD	*A*	*K*	α	1	2	3	4
1. Concern about a variant	4.38	4.38	1.01	0.18	0.89	−			
2. Religious fatalism	15.68	6.45	0.21	-0.43	0.91	0.14**	−		
3. Vaccine acceptance	14.33	4.11	-1.02	0.69	0.92	0.20**	0.25**	−	
4. Vaccine rejection	22.73	7.40	-0.26	-0.89	0.70	0.16**	-0.45**	0.12**	–

M, mean; SD, standard deviation; A, asymmetry; K, kurtosis; α, alpha coefficient; **significance at 0.01.

**FIGURE 1:**
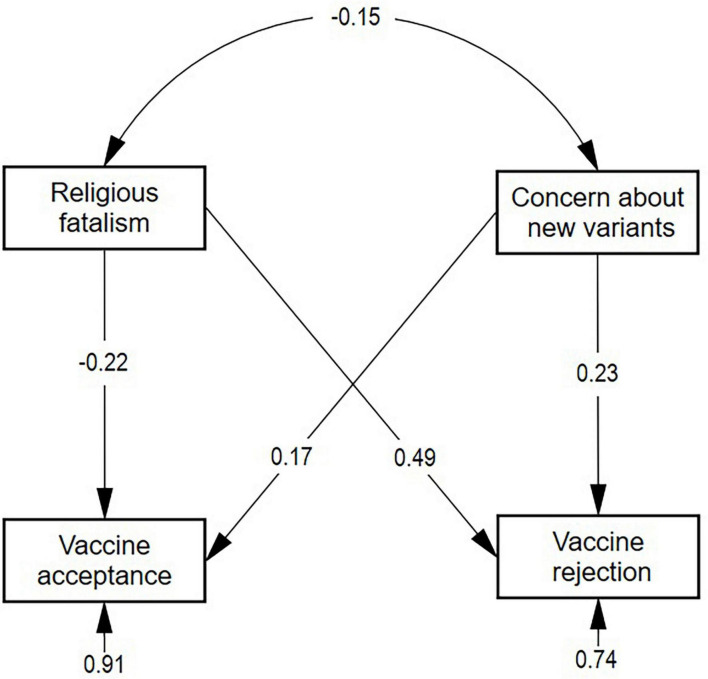
Results of the explanatory structural model of vaccine acceptance. The standardized estimated parameters are shown, where F1 is religious fatalism, F2 is concern about new variants, S_vcns is vaccine acceptance, and N_vcns is vaccine rejection.

The analysis of the proposed model showed that an adequate fit was obtained, χ^2^ = 139.10, *p* = 0.000, CFI = 0.998, TLI = 0.987, GFI = 0.998, NFI = 0.992, RMSEA = 0.029, 95% CI [0.048–0.06], SRMR = 0.043. Based on these results (Figure 1), H1 is confirmed since a negative effect of religious fatalism on the acceptance of vaccines is observed (β = −0.22, *p* < 0.001); H3 is confirmed since a positive effect of fatalism is observed on the concern about new variants (β = −0.49, *p* < 0.001); and H4 is confirmed since a positive effect of concern about new variants on vaccine acceptance is observed (β = −0.17, *p* < 0.001). On the other hand, in terms of H2, there is an effect of concern about new variants on the vaccine rejection (β = −0.23, *p* < 0.001), which is positive, contrary to what was proposed.

## 4. Discussion

Amid the COVID-19 pandemic, vaccination has been shown to reduce the harmful effects of COVID-19 on human health; therefore, an increase in its acceptance is expected to prevent the negative effects of the new variants that are emerging. For this reason, it is important that the population develop favorable attitudes toward vaccination with a view to mitigating infection. Accordingly, the present study sought to determine whether religious fatalism and concern about new variants affect the acceptance of COVID-19 vaccines.

Religious fatalism had a negative influence on vaccination against new variants, similar studies indicated that rejection in religious groups is due to the fact that vaccines tend to be an act of interfering with divine providence (12, 39). That is, to the extent that conflicting religious beliefs about the origin and consequences of COVID-19 prevail, the less likely vaccine acceptance will be. This result agrees with what was revealed by Justin and Vaidyanathan (40), who, after studying 12 congregations (Buddhist, Christian, Hindu, Jewish, and Muslim) in the United States, concluded that religiosity is negatively associated with vaccine acceptance. Similarly, a qualitative study conducted with Orthodox Jews from the United Kingdom and Israel found that the discourse of religious exemption and opposition impaired the intention to be vaccinated (41).

Despite the relevance of the finding, it is important to clarify that not all religious organizations are anti-vaccination since some religious groups argue that the vaccine is a gift from God (42), and their religious authorities support vaccination and reject the marginal perspectives that reject it, clarifying that vaccination does not violate their beliefs since it seeks the preservation of health (43). Even historically, some religions rejected the spread of diseases because their sacred books promoted hygiene such as distancing or quarantine for some contagious diseases, hand washing and other medical advice (44). Given this context, we believe that religious education can promote protection against chronic diseases such as SARS-CoV-2 and prevent their spread (26). Thus, it is important to identify religion as a key component in decisions; although fundamental beliefs are difficult to change, it is necessary to associate them with religious and community leader-ship to promote greater acceptance of vaccines (45).

On the other hand, concern for a new variant of COVID-19 was positively associated with vaccination. This result differs from what was reported by previous studies (46, 47), which indicate that rejection or hesitation to vaccination tends to increase with concern for the vaccine. This leads to understanding that the greater the concern for infection from a new variant, the more likely is the rejection of the vaccine. Leading to the understanding that the greater the concern for infection from a new variant is, the more likely vaccine rejection is. In this regard, previous studies have indicated that vaccination increased amid the appearance of some variants (48). Therefore, the result would contradict what has been reported in the scientific literature. However, it is necessary to take into account the behavior of some variables in this study. For example, the average scores for religious fatalism and vaccine rejection were 15.68 (from a minimum of 6 to and a maxi-mum of 30), and 22.73 (from a minimum of 8 and a maximum of 40), which exceed the cutoff of 50. This fact would help us understand that in the majority of participants, religious fatalism and vaccine rejection are predominant, which would influence the functional relationship between these variables.

In addition, it is necessary to recognize that although vaccines are associated with decreased viral load, their efficacy tends to decrease with the emergence of new variants; therefore, the population may have doubts founded on decreased immunity (49). On the other hand, even though the transmission of the virus persists in vaccinated populations, 44 variants have the ability to evade antibodies and transmit diseases in an improved way (50). Therefore, they require different precautions, such as monitoring the transformation of the virus through increased genomic surveillance and attitudes toward the pandemic. Given this insight, the scientific community has recommended booster doses, which have been shown to generate significant immunity against COVID-19 variants (51).

Likewise, religious fatalism had a positive influence on vaccine acceptance, this is because other religious groups argue based on the Bible that the vaccine is a gift from God (41). Similar studies have indicated that in religious groups, rejection arises because vaccines tend to represent an interference with divine providence (12). Thus, some religious groups believe that inoculation interferes with divine will since God does not allow diseases to occur, and thus, vaccination represents distrust of a higher being (42). It is important to evaluate religious factors since they prevent equitable access to vaccination and increase the risk of contracting COVID-19. In this way, religious leaders challenge the implementation of the closure of places of religious activity such as churches or sanctuaries, leading to a rapid increase in infection rates; additionally, they spread conspiracy theories claiming that the vaccines are “infidel vaccines” and that they go against their identity, beliefs and religious practices (52–54). In addition, religions that prohibit pork claim the religious deviance of the vaccine by the use of pork gelatin or tissues from human fetuses in experimentation, making the vaccines impure or religiously unlawful (44). On the other hand, it has also been suggested that vaccination is unnecessary because there are other alternatives, such as pharmacological treatments or prevention, including trust in spirituality and prayers (26). Therefore, individuals with high levels of conflictive spirituality tend to reject the vaccine based on apocalyptic ideas and defend alternative medicine (55). Finally, lack of knowledge in religious groups regarding the safety and efficacy of the new vaccine developed to combat the new coronavirus decreases the probability of vaccination (56).

Concern for a new variant of COVID-19 was negatively associated with vaccination. Thus, it is assumed that the concern for infection by a new variant is positively associated with vaccine acceptance. Although some similar studies indicate high rates of reluctance to vaccination in groups concerned about the safety of the vaccine (57–59), it is possible that perceptions and attitudes change rapidly since greater efficacy and reduced side effects are expected (12, 60). Accordingly, people agree to receive the vaccine if they recognize the advantages related to its use (61). However, the inconsistent information provided by political leaders contrasts with expert voices on vaccination issues. Additionally, vaccine technology is not familiar to the majority of the population, which may explain increased concern about COVID-19 vaccines (56). Meanwhile, it is necessary to receive all the doses of the vaccines to be completely immunized against infection (62). At this point, what is important to highlight is that in-creased awareness of severity, mortality and susceptibility enables an increase in the intention to adopt prevention measures (63). Therefore, it is important to provide information on vaccine efficacy and develop strategies to overcome concerns about vaccines and promote vaccine safety despite the accelerated development of this vaccine (64).

### 4.1. Implications

Our results can help to illuminate the factors that influence the dynamics of the development of vaccine acceptance, especially in developing countries where the illiteracy rate is high and people do not understand the science behind vaccine development. Therefore, communications and a greater openness to the needs of communities and the concerns of the people in that community are solid strategies to address vaccine rejection. In this case, health professionals must provide safe and reliable information to the religious community so that religious scholars can base their views on religious arguments and raise awareness of conspiracy theories. Likewise, efforts by the state, health professionals and religious leaders through social campaigns that promote vaccine acceptance by highlighting the usefulness of vaccines, are necessary.

### 4.2. Limitations

First, a cross-sectional design was adopted. Therefore, the capacity to determine causal inferences is limited between the predictors and the perception of vaccine acceptance. Second, the vaccine acceptance rate can fluctuate according to the current situation concerning the pandemic. Third, non-probabilistic sampling does not allow generalization of the results to the general population. Fourth, the fact that samples were not proportionately similar in gender and age may limit the scope of the results of this research, therefore, in future studies, it would be necessary to analyze by gender and age. Fifth, there is likely to have been a bias in the selection of the sample, since the sampling procedure was not random. Finally, it is also necessary to mention the possibility of the existence of confounding factors, through variables that distort the measure of the association between the other studied variables. Despite these limitations, an increase in information exchange and communication by health workers and the state with religious leaders/academics will reduce doubts about vaccination against SARS-CoV-2 variants and will promote greater dissemination of knowledge about the disease.

## 5. Conclusion

It is concluded that religious fatalism has shown a negative effect on the acceptance of the SARS-CoV-2 vaccine. Despite the fact that in scientific literature religious practices are interpreted as determinants of health, conflicting religious beliefs about the origin and consequences of COVID-19 have clearly generated distrust in the effectiveness of vaccines. On the other hand, the emergence of new variants has had a significant effect on the intention to vaccinate against COVID-19, as manifestations of uncertainty, fear, and fear of the new effects that they could cause have sensitized the general population so that they can protect themselves from adverse effects.

## Data availability statement

The raw data supporting the conclusions of this article will be made available by the authors, without undue reservation.

## Ethics statement

The studies involving human participants were reviewed and approved by the Ethics Committee of the Universidad Peruana Unión (N° 2022-CEUPeU-025). The patients/participants provided their written informed consent to participate in this study.

## Author contributions

OM-B, RF-S, and ET conceived and designed the experiments, performed the experiments, analyzed and interpreted the data, and wrote the manuscript. MH-M, MT-B, and WM-G contributed to the reagents, materials, analysis tools or data, and wrote the manuscript. All authors contributed to the article and approved the submitted version.
